# Factors predicted quality of life of people with type 2 diabetes in western Ethiopia

**DOI:** 10.1371/journal.pone.0281716

**Published:** 2023-02-15

**Authors:** Dereje Chala Diriba, Doris Y. P. Leung, Lorna K. P. Suen

**Affiliations:** 1 School of Nursing, The Hong Kong Polytechnic University, Hong Kong, Hong Kong SAR, China; 2 School of Nursing, Tung Wah College, Hong Kong, Hong Kong SAR, China; University of Almeria: Universidad de Almeria, SPAIN

## Abstract

**Background:**

Multiple factors predict the quality of life of adults with diabetes. However, the relationships of demographics, self-management practice, and support status with the quality of life of people with diabetes are unknown. Therefore, the study aimed to assess factors related with the quality of life of adults with type 2 diabetes in western Ethiopia.

**Methods:**

A hospital-based cross-sectional study involving adults with type 2 diabetes was conducted in western Ethiopia from June 02, 2020, to August 31, 2020. Convenience sampling technique was used in selecting subjects. The translated and psychometrically tested summary of diabetes self-management activities (expanded), diabetes quality of life, and diabetes care profile support scales were used in measuring self-management practice, quality of life, and support status, respectively. Data were collected via face-to-face interviews. Factors related with quality of life were examined through bivariate analysis and multivariable linear regression. In all statistical tests, P value <0.05 and confidence level that excluded zero were considered statistically significant.

**Results:**

A total of 417 adults with type 2 diabetes participated in the study. In a multivariable linear regression, seven factors including age, male, homemakers, those separated/divorced, number of years since diabetes diagnosis, self-management practice and support needed were related with quality of life. Male patients (β = 2.786, 95% CI = 1.285 to 4.287, p < 0.001), homemakers (β = 0.366, 95% CI = 0.056; 0.677, p = 0.021), self-management practice (β = 4.528, 95% CI = 3.851 to 5.205, p < 0.001) and those who needed support from their families or peers (β = 1.623, 95% CI = 0.458; 2.788, p = 0.006) were related positively with quality of life whereas those who separated or divorced (β = −1.698, 95% CI = −3.371 to −0.025, p = 0.047), older age (β = −0.195, 95% CI = −0.269 to −0.121, p < 0.001) and those who lived with diabetes for a longer duration (β = −2.206, 95% CI = −4.151 to −0.261, p = 0.026) were related negatively with quality of life.

**Conclusion:**

Quality of life of people with type 2 diabetes living in western Ethiopia was predicted positively by being male, homemakers, having self-management practice, and support needed, whereas negatively influenced by old age, separation or divorce, and long diabetes life. Thus, encouraging self-management practice, and continuous family or friend support are necessary to enhance quality of life of people with type 2 diabetes. Further study should employ random sampling techniques and involve participants from multiple study settings to increase representativeness of the samples.

## Introduction

Diabetes is a serious global health threat, showing rapid increase in prevalence annually [1,2]. According to the International Diabetes Federation Atlas report, in 2021, more than half a billion adults globally and more than 24 million in Africa have diabetes. Ethiopia has the fourth largest number of diabetes cases in Africa [1].

Diabetes has health, economic, social, and psychological impacts [3]. The World Health Organization (WHO) reported that the risk of developing medical conditions like heart attacks, stroke, blindness, kidney failure, and neuropathy increases two- to threefold among adults with diabetes. Moreover, people with diabetes risk have a reduced quality of life (QOL) [4]. It is necessary to improve QOL among people with diabetes, and WHO set a target for all countries to secure the best QOL by 2025 [2]. Diabetes specific QOL has four domains, including satisfaction, impact, social or vocational worry, and diabetes-related worry [5].

Quality of life is concerned with the psychological well-being, psychological care, and experience of patients and recognized as an essential aspect of a patient’s health; however, it is rarely assessed in diabetes research [6]. The WHO defines QOL as “individuals’ perceptions of their position in life in the context of culture and value systems in which they live and in relation to their goals, expectations, standards, and concerns” [7]. QOL is a significant predictor of premature mortality [8,9]. Given the high cost of diabetes care, shortage in workforce, low access to health information systems and supply chains, and poor service delivery, the quality of diabetes care in sub-Saharan Africa is poor [10,11]. Diabetes care in sub-Saharan Africa is poor, and thus QOL remains poor [11]. Poor glycemic control is related with poor quality of life [12].

Although QOL is rarely assessed in people with diabetes [13], few studies have assessed QOL [14–18]. Medium-level or better QOL was reported in people with diabetes in Iran and Hong Kong [16,18] compared with people with diabetes in Ghana and Nigeria [19]. Medium-level QOL was reported in people with type 2 diabetes in Ethiopia [20]. These studies reported different factors related to the QOL of people with diabetes. Gender, marital status, multimorbidity, age, employment status, level of education, inactive lifestyle, and receiving insulin treatment were found to be significantly related with QOL [16,19–23]. In addition, living with diabetes-related complications is related to poor QOL [9]. However, a broad range of potential factors related with QOL, such as self-management practice and social support from family or friends, has not been assessed in people with diabetes.

In addition, QOL in some of these studies, including those conducted in Ethiopia, was measured with generic tools that are not specific to diabetes [17,20,23]. Disease-specific tools for measuring QOL are generally recommended, such as the diabetes quality of life (DQOL) tool, for people with diabetes [24,25]. Thus, this study aimed to identify factors related with QOL in adults with type 2 diabetes in western Ethiopia from a broad range of factors by using a disease-specific QOL measure.

## Methods

### Study design and setting

A hospital-based cross-sectional study was employed from June 01, 2020, to August 31, 2020. Data were collected from Nekemte Specialized Hospital, western Ethiopia. The hospital is located in Nekemte, 331 km west of the capital city of Ethiopia; a total of 75,219 people were living in Nekemte in 2007 [26]. The hospital is a comprehensive public hospital that provides different health services, including diabetes care, surgical, medical, obstetric, gynecologic, pediatric, and neonatal care services. The catchment population of the hospital is 3.5 million people and is a center of referral service for 10 million people in western Ethiopia [27]. People with diabetes receive healthcare in the diabetes center in the hospital and undergo monthly follow-up. According to the hospital source, the center is staffed with one physician and three nurses.

### Participants

Data collectors approached people with diabetes in the hospital. Subjects were included if they 1) had been diagnosed with type 2 diabetes, 2) had been treated at the diabetes center in the participating hospital for six months or more, 3) were in a stable medical condition (those who have no acute illness), 4) were aged 18 or over, 5) were cognitively intact (as determined by their medical records), and 6) were able to speak and understand Afaan Oromoo (a local language). They were excluded if they 1) refused to participate in the study, 2) had a hearing problem, or 3) were from prison custody because they would not have adequate time to provide their responses.

### Sample size calculation

The required sample size was estimated based on multiple linear regression using G*Power 3.0. Since previous studies in Ethiopia did not report R^2^ [20,28,29], to be conservative, we assumed a small effect size of R^2^ = 0.04 with 10 predictors in the final regression model, a sample size 417 is required to achieve a 80% power with a significance level of 5%.

### Sampling technique

A convenience sampling technique was used in selecting participants for the study from people with type 2 diabetes attending the diabetes center of the hospital in western Ethiopia for their monthly medical follow-up.

### Variables

i) Dependent variable
  Quality of life.
ii) Independent variables

Sociodemographic variables, including age, gender, marital status, ethnicity, religion, education level, primary caregiver, employment status, and patient-related factors, such as the status of the diabetes-related disease, type of diabetes-related disease, and number of years since diabetes diagnosis was performed. In addition, self-management practice, support needed, support received, and support attitude were collected.

### Data collection tools

The questionnaire was developed and includes sociodemographic characteristics and three adopted and psychometrically tested scales, including diabetes quality of life, summary of diabetes self-care activities (expanded), and diabetes care profile–support scale.

#### a) Diabetes quality of life

Quality of life is the level of an individual’s perception of their position in life in the context of the culture and value systems in which they live and in relation to their goals, expectations, standards and concerns [7]. It was assessed using the diabetes quality of life-Afaan Oromoo measure [30]. The tool has 34 items covering four domains: satisfaction (13 items), impact (13 items), social or vocational worry (5 items), and diabetes-related worry (3 items). All items were evaluated using a five-point scale (1–5), and the scores range from 1 (very satisfied) to 5 (very dissatisfied) for satisfaction. Items in the impact and the two worry domains were scored also with a five-point scale, and the scores range from 1 (no impact and never worried) to 5 (always impacted and always worried). If an item was not appropriate to the respondent in the social or vocational worry and diabetes-related worry subscales, the “Does not apply” option was provided, and the corresponding item was not scored. The scores were arithmetically transformed from a 0 (the lowest possible QOL) to a 100 (the highest possible QOL) score [31,32] for easy interpretation, and a high score indicated good QOL. A high score in the satisfaction domain indicated the satisfaction with diabetes life; however, the high scores in impact, social or vocational worry, and diabetes-related worry indicated low impact and reduced worry. The Cronbach’s alpha value was 0.867 for the overall DQOL-AO, 0.846 for the satisfaction, 0.827 for the impact, 0.654 for the social/vocational worry, and 0.727 for the diabetes-related worry domains [30].

#### b) Support status

Three types of support from family or friends were measured, including diabetes care profile–support needed, support received, and support attitudes by using the scales developed to measure social support provided for people with diabetes by the Michigan Diabetes Research Center [33]. Support needed refers to a level of a need of support from either family or friends who can potentially help a participant during illness that could be emotional or instrumental or both [34,35]. Support received refers to the level of received the emotional or instrumental support from families or friends [35]. The support needed scale and support received scale measure the support needed and support received to plan diet, medicine intake, foot care, physical activity, blood glucose testing, and sick day management. Support attitudes is the level of perceived patient’s attitude towards support attitudes by families or friends about diabetes and self-care, expectations for medical management and outcomes [36]. The support attitudes scale measures a family’s or friend’s attitudes to supporting people with diabetes. These three scales measure the perceptions of diabetic patients. Each scale has six items and is scored on a five-point Likert scale (strongly disagree [1] to strongly agree [5]). For each scale, an average score was computed according to corresponding items and can range from 1 to 5 (high scores indicated good support in each support type, support needed, support received, and support attitudes). These scales showed acceptable to excellent reliability for African Americans, ranging from 0.70 to 0.97 [37]. The Afaan Oromoo versions of the scales showed acceptable to good reliability, ranging from 0.706 to 0.809 in the current study.

#### c) Self-management practice

Self-management practice refers to ‘the practical ability of a patient to deal with chronic illnesses, including symptoms, treatment, physical and social consequences and lifestyle changes’ in a week [38]. Self-management practice for diabetes care was measured using the Summary of Diabetes Self-Care Activities-Expanded scale (SDSCA) [39], which is a patient-reported measure covering self-management practice for diet, physical activity, medication, foot care, and blood glucose testing behavior and rates the number of days the people with diabetes had practiced these care activities for last 7 days. Ten SDSCA-Afaan Oromoo version items were obtained after the scale’s correlation was tested and exploratory factor analysis was performed. The scale has 10 items, with internal consistency of 0.730, which indicates acceptable reliability. Scores ranged from 0 to 7. The mean score of days of self-management practiced was calculated, and high scores indicated good self-management practice.

### Data collection techniques

The data were collected through face-to-face interviews. A one-day training workshop was conducted to enable data collectors to familiarize themselves with the items on the scale and the methods for conducting interviews. People with diabetes were contacted when they were waiting to see a doctor in the diabetes center of the hospital. After explaining the purpose and risks of the study, the data collectors assessed the people with diabetes for their eligibility. Having obtained their informed written consent, the data collectors then administered the questionnaire.

### Statistical methods

The statistical analyses were performed using SPSS version 25. Descriptive statistics were calculated for sociodemographic characteristics, quality of life, self-management practice, support needed, support received, and support attitudes. The frequency and percentage were calculated for categorical variables, whereas mean and standard deviation were calculated for continuous data. Model assumptions, including normality, linearity, multicollinearity, and equal variance, were checked. Bivariate analyses of overall QOL and its four domains with other variables were conducted using a) the Pearson product-moment correlation for continuous variables like age, year since diabetes diagnosis, self-management practice, support needed, support received and support attitudes, b) independent samples t-test for binary variables, including gender, ethnicity and primary caregiver, and c) ANOVA for categorical variables with three or more options that entails religion, marital status, education level, employment status, and diabetes comorbid disease. The assumptions for MANOVA test were not fulfilled for domains; hence, separate ANOVAs were conducted. Multivariable analyses were conducted with linear regression model using variables that were significant in bivariate analyses. The mean score of QOL was determined using Tukey’s post hoc test for variables with three or more categories, and independent samples t-test was used in estimating mean for variables with two categories. A p-value <0.05 and confidence interval excluded zero in bivariate and multivariable analyses was considered statistically significant.

### Ethical considerations

Ethical approval was obtained from the human subjects ethics subcommittee of The Hong Kong Polytechnic University (Reference number: HSEARS20200317007) and Wollega University Institutional Review Board (Reference number: ወ/ዩ 165,429/D1-2). Permission to collect the data was obtained from the participating hospital, and informed written consent was obtained from each participant. Each completed questionnaire was coded, and the code was used in data entry to ensure the confidentiality of the response.

## Results

### Sociodemographic characteristics of the participants

A total of 417 participants responded to all items. The mean age was 50.2 ± 11.7 years. Half of the participants (51.3%) were females, and a quarter (24.7%) of the subjects had attended tertiary education. Slightly more than a quarter (28.5%) were employed, and nearly two-thirds (62.1%) received support from their spouses. Hypertension was the most common comorbid disease (45.6%). The mean number of years since diabetes diagnosis was 6.2 ± 4.5 years (Table 1).

**Table 1:** Sociodemographic characteristics, quality of life, self-management practice, and support status of the participants (n = 417).

Variables	Categories	Frequency (%) / Mean ±SD
Age	Age (in years)	50.2 ± 11.7
Gender	Female Male	214 (51.3%) 203 (48.7%)
Marital status	Married Widowed Never married Separated/divorced	323 (77.5%) 56 (13.4%) 30 (7.2%) 8 (1.9%)
Ethnicity	Oromoo Amhara	368 (88.2%) 49 (11.8%)
Religion	Christian (Protestant) Christian (Orthodox) Muslim	237 (56.8%) 138 (33.1%) 42 (10.1%)
Education level	No formal education Elementary school Secondary school Tertiary education	76 (18.2%) 138 (33.1%) 100 (24.0%) 103 (24.7%)
Employment status	Unemployed Employed Homemaker	241 (57.8%) 119 (28.5%) 57 (13.7%)
Primary caregiver	Spouse Child	259 (62.1%) 158 (37.9%)
Diabetes comorbid disease	No comorbid disease Hypertension Other diseases**	186 (44.6%) 190 (45.6%) 41 (9.8%)
Year since diabetes diagnosis	Years since diagnosis with diabetes	6.2 ± 4.5
Quality of life	Overall QOL score Satisfaction Impact Social/vocational worry Diabetes-related worry	64.79 ± 9.09 70.36 ± 10.53 71.72 ± 10.09 37.66 ± 20.29 55.84 ± 13.09
Self-management practice	Self-management practice (days per week)	2.98 ± 1.05
Support status	Support needed Support received Support attitudes	4.71 ± 0.41 3.74 ± 0.76 4.61 ± 0.47

**neuropathy, hyperlipidemia, valvular heart disease.

### Quality of life, self-management practice, and support status

The mean score of QOL was 64.79 ± 9.09. The higher QOL score was attributed to impact domain (71.72 ± 10.09) and satisfaction domain (70.36 ± 10.53), whereas lower QOL score resulted in social/vocational worry (37.66 ± 20.29). The mean number of days on which people with type 2 diabetes practiced self-management per week was 2.98 ± 1.05. Adults with type 2 diabetes support needed was 4.71 ± 0.41 and support attitudes was 4.61 ± 0.47 from their respective families or peers. However, the support received was 3.47 ± 0.76, as shown in Table 1.

### Factors related with quality of life and its domains

The bivariate analyses results showed that age (r = 0.282, p < 0.001), gender (t = 1.980, p = 0.048), marital status (F = 4.600, p = 0.004), education status (F = 11.966, p < 0.001), employment status (F = 13.600, p <0.001), types of comorbidity (F = 7.603, p < 0.001), year since diabetes diagnosis (r = −0.168, p < 0.001), primary caregiver (t = 3.510, p < 0.001), self-management practice (r = 0.549, p < 0.001), support needed (r = 0.184, p < 0.001), and support received (r = 0.164, p = 0.001) were related with quality of life (Table 2). These variables were used in the multivariable analyses.

**Table 2:** Bivariate and multivariable analyses of variables with overall quality of life.

Variables	Quality of life			
	Mean (SD)	Bivariate analysis	Multivariable analysis	
		Test statistics (p-value)	β- value (95% CI)	p-value
Age		0.282^a^ (<0.001)	−0.195 (−0.269; − 0.121)	**<0.001**
Gender Female* Male	63.93 (8.37) 65.69 (9.74)	1.980^b^ (0.048)	2.786 (1.285; 4.287),	**< 0.001**
Marital status Married* Widowed Never married Separated/divorced	65.18 (8.74) 61.76 (10.19) 67.78 (8.26) 58.82 (11.78)	4.600 ^c^ (0.004)	0.367 (−0.241; 0.975) 1.161 (−0.711; 4.032) −1.698 (−3.371; −0.025)	0.236 0.427 0.047
Ethnicity Oromoo Amhara	64.98 (9.31) 63.36 (7.18)	1.170^b^ (0.242)		
Religion Protestant Christian Orthodox Christian Muslim	65.63 (9.72) 63.79 (7.68) 63.35 (9.41)	2.396^c^ (0.092)		
Education level No formal education* Elementary school Secondary school Tertiary education	62.25 (9.48) 62.89 (8.15) 65.08 (9.22) 68.92 (8.51)	11.966^c^ (<0.001)	−0.309 (−1.350; 0.731), 0.011(−0.746; 0.768), 0.547 (−0.074; 1.168),	0.559 0.977 0.084
Employment status Unemployed* Employed Homemaker	63.14 (9.37) 68.29 (8.71) 64.46 (6.36)	13.600^c^ (< 0.001)	1.244 (−0.669; 3.156) 0.366 (0.056; 0.677)	0.202 **0.021**
Types of diabetes comorbid disease No comorbid disease* Hypertension Other comorbid diseases**	66.57 (8.71) 63.74 (9.41) 61.59 (7.75)	7.603^c^ (0.001)	−0.643 (−2.231; 0.945) −0.209 (−1.055; 0.636)	0.426 0.627
Year since diabetes diagnosis		-0.168^a^ (<0.001)	−2.206 (−4.151; −0.261)	**0.026**
Primary caregiver Spouse* Child	65.99 (8.33) 62.82 (9.94)	3.510^b^ (<0.001)	−0.195 (−0.487; 0.097)	0.190
Self-management practice		0.549^a^ (<0.001)	4.528 (3.851; 5.205)	**<0.001**
Support needed		0.184^a^ (<0.001)	1.623 (0.458; 2.788),	**0.006**
Support received		0.164^a^ (0.001)	0.552 (−0.412; 1.515),	0.261
Support attitudes		−0.080^a^ (0.102)		

^a^ Pearson correlation

^b^ independent samples t-test statistics

^c^ ANOVA F-statistics

β: Linear regression coefficient

*Reference category

**neuropathy, hyperlipidemia, valvular heart disease; the bold font in the P-value indicate statistically significant findings.

Seven variables: age, gender, employment status, marital status, years since diabetes diagnosis, self-management practice, and support needed remained statistically significant in the multivariable analysis of quality of life, and showed 48.5% variability (R^2^ = 0.485, p < 0.001). Being male (β = 2.786, 95% CI = 1.285 to 4.287, p < 0.001), homemakers (β = 0.366, 95% CI = 0.056 to 0.677, p = 0.021), those who practiced diabetes self-management practice (β = 4.528, 95% CI = 3.851 to 5.205, p < 0.001) and who needed support from their families or peers (β = 1.623, 95% CI = 0.458 to 2.788, p = 0.006) were related positively with QOL in adults with type 2 diabetes. Those who were separated/divorced (β = −1.698, 95% CI = −3.371 to −0.025, p = 0.047), old age (β = −0.195, 95% CI = −0.269 to −0.121, p < 0.001) and living with diabetes for a long time (β = −2.206, 95% CI = −4.151 to −0.261, p = 0.026) were related negatively with quality of life (Table 2).

The bivariate analyses showed that age related with satisfaction, impact, social/vocational worry, and diabetes-related worry domains. Marital status related with satisfaction, impact, and diabetes-related worry domains in bivariate analyses. While education level and employment status related with all domains of QOL, religion only related with social or vocational worry domain. Type of comorbid disease related with social or vocational worry and impact domains. The duration of diabetes diagnosis negatively correlated with satisfaction, impact, and social/vocational worry domains. Primary caregiver only related with impact, social or vocational worry and diabetes-related worry domains. Self-management practice positively correlated with all domains, support needed positively correlated with satisfaction, impact, and social or vocational worry domains. However, those who received support from families or peers positively correlated with satisfaction and diabetes-related worry domains. Those who had support attitudes negatively correlated with satisfaction domain.

The multivariable analyses using statistically significant variables in bivariate analyses with domains of quality of life indicated the variance (R^2^) ranging from 17.9% to 37.5%. The findings showed that old age was related negatively with satisfaction, impact, social or vocational worry, and diabetes-related worry. Male patients with type 2 diabetes related negatively with social or vocational worry compared with females with the same disease. Widowed patients related positively with satisfaction, those separated or divorced were related negatively with satisfaction compared with those married. Diabetic patients with hypertension negatively predicted satisfaction and had long diabetes life were related negatively with social or vocational worry. Those who practiced good diabetes self-management related positively with satisfaction, impact, worry because of social issues, and worry due to diabetes. Those who needed family or peer support related positively with diabetes impact, and those who received a support from their family or peer related positively with satisfaction and worry due to diabetes. Having a support attitude from their family or peer was related negatively with satisfaction (Table 3).

**Table 3:** Bivariate and multivariable results of variables with QOL domains.

Variables	Quality of life domains							
	Satisfaction		Impact		Social/vocational worry		Diabetes-related worry	
	Bivariate analysis	Multivariable analysis	Bivariate analysis	Multivariable analysis	Bivariate analysis	Multivariable analysis	Bivariate analysis	Multivariable analysis
	Test statistics (p-value)	β (95% CI), p-value	Test statistics (p-value)	β (95% CI), p-value	Test statistics (p-value)	β (95% CI), p-value	Test statistics (p-value)	β (95% CI), p-value
Age	−0.155 ^a^ (0.001)	−0.136 (−0.225; −0.046), 0.003	−0.256 ^a^ (<0.001)	−0.211 (−296; −0.126), <0.001	−0.264^a^ (<0.001)	−0.369 (−548; −0.191), <0.001	−0.142^a^ (0.004)	−0.145 (−0.270; −0.021), 0.022
Gender Female* Male	1.060 ^b^ (0.290)		0.386^b^ (0.699)		3.038^b^ (0.003)	7.703 (3.784; 11.623), <0.001	2.795^b^ (0.005)	2.513 (−0.124; 5.150), 0.062
Marital status Married* Widowed Never married Separated/divorced	2.835^c^ (0.038)	0.711(0.052; 1.370), 0.035 0.567 (−2.776; 3.909), 0.739 −2.102 (−4.084; −120), 0.038	3.439^c^ (0.017)	0.018 (−0.706; 0.741), 0.962 0.630 (−2.809; 4.069), 0.719 −1.828 (−3.833; 0.177), 0.074	2.464^c^ (0.062)		5.059^c^ (0.002)	−0.491 (−1.567; 0.586), 0.371 0.308 (−4.710; 5.325), 0.904 −0.805 (−3.768; 2.158), 0.594
Ethnicity Oromoo* Amhara	1.401^b^ (0.162)		0.932^b^ (0.352)		0.549^b^ (0.583)		0.197^b^ (0.844)	
Religion Protestant Christian* Orthodox Christian Muslim	0.649^c^ (0.523)		2.355^c^ (0.096)		4.582^c^ (0.011)	−1.480 (−3.485, 0.526), 0.100 −0.700 (−2.961, 1.561), 0.169	1.180^c^ (0.308)	
Education level No formal education* Elementary school Secondary school Tertiary education	10.173^c^ (<0.001)	−0.310 (−1.572; 0.953), 0.630 0.224 (−0.691; 1.140), 0.630 0.438 (0.300; 1.177), 0.244	4.751^c^ (0.003)	−0.284 (−1.504; 0.936), 0.647 −0.162 (−1.026; 0.702), 0.713 0.237 (−0.459; 0.932), 0.504	4.174^c^ (0.006)	−0.4791 (−3.176; 2.219), 0.737 −0.709 (−2.649; 1.230), 0.444 0.248 (−1.321; 1.817), 0.770	14.492^c^ (<0.001)	0.427 (−1.378; 2.231), 0.642 0.353 (−0.961; 1.667), 0.597 1.636 (0.558; 2.713), 0.003
Employment status Unemployed* Homemaker Employed	10.373^c^ (<0.001)	0.164 (−0.198, 0.525), 0.374 1.051(−1.265, 3.367), 0.373	7.016^c^ (0.001)	0.174 (− 0.175, 0.524), 0.328 0.463 (−1.780, 2.705), 0.685	8.453^c^ (<0.001)	0.584 (−0.217, 1.385), 0.124 3.545 (−1.439, 8.528), 0.153	5.062^c^ (0.007)	0.152 (− 0.393, 0.696), 0.584 −0.762 (−4.135, 2.611), 0.657
Types of diabetes comorbid disease No comorbid disease* Hypertension Other comorbid diseases**	6.264^c^ (0.002)	−2.014 (−3.950; −0.078), 0.041 −0.354 (−1.382; 0.674), 0.499	7.945^c^ (<0.001)	0.322 (−1.527; 2.171, 0.732 −0.911 (−1.922; 0.099), 0.685	2.560^c^ (0.079)		2.740^b^ (0.066)	
Years since diabetes diagnosis	-0.098^a^ (0.045)	−2.079 (−4.441; 0.282), 0.084	−0.122^a^ (0.013)	−1.211 (−3.516; 1.094), 0.302	−0.202^a^ (<0.001)	−6.526 (−11.553; −1.500), 0.011	0.051^a^ (0.297)	
Primary caregiver Spouse* Child	1.655^b^ (0.099)		2.973^b^ (0.003)	−0.179 (−0.524; 0.165), 0.306	3.417 ^b^ (<0.001)	−0.226 (−0.081; 0.429), 0.501	−2.983^b^(0.003)	−0.128 (− 0.640; 0.384), 0.623
Self-management practice	0.510^a^ (<0.001)	4.675 (3.855; 5.494), <0.001	0.501^a^ (<0.001)	4.406 (3.610; 5.201), <0.001	0.273^a^ (<0.001)	4.988 (3.219; 6.757), <0.001	−0.173^a^ (<0.001)	1.354 (0.183; 2.524), 0.024
Support needed	1.000^a^ (0.042)	0.142 (−1.268; 1.552), 0.843	0.231^a^ (<0.001)	2.572 (1.182; 3.962), <0.001	0.123^a^ (0.012)	3.002 (−0.081; 6.085), <0.054	0.014^a^ (0.782)	
Support received	0.321^a^ (<0.001)	3.076 (−4.984; −1.259), <0.001	−0.011^a^ (0.832)		−0.028^a^ (0.194)		0.285^a^ (<0.001)	3.829 (2.142; 5.517) (<0.001)
Support attitudes	−0.150 ^a^ (0.002)	−4.621 (− 4.984; − 1.259), 0.007	−0.450 ^a^ (0.355)		0.038 ^a^ (0.444)		−0.058^a^ (0.241)	

^a^ Pearson correlation

^b^ independent samples t-test statistics

^c^ ANOVA F-statistics

β: Linear regression coefficient

*Reference category

**neuropathy, hyper lipidaemia, valvular heart disease.

## Discussion

This study examined factors related with quality of life in adults with type 2 diabetes in western Ethiopia. Our study found being male, homemakers, self-management practice, and support needed related positively with QOL. However, old age, separation or divorce, and living with diabetes for a long duration were predicted negatively quality of life. Self-management practice related positively with all the four domains of QOL. Support needed was related positively with impact and social or vocational worry. Receiving support from family or peer related positively with satisfaction and diabetes-related worry. However, support attitude related negatively with satisfaction. Age negatively related with all domains. Being male related positively with social or vocational worry.

Consistent with previous studies in Bangladesh, Iran, Indonesia, and Taiwan [15,16,40,41], our study found that self-management practice was positively related with overall QOL and all its domains after the effects from other related factors in the multivariable analyses were controlled. Self-management practice depends on knowledge, decision-making capacity, and bearing the necessary skills [3], and effective self-care promotes a healthy lifestyle and improves QOL [42]. Given the consistent findings in self-management practice in QOL in people with diabetes, self-management behavior should be encouraged, and QOL should be enhanced through diabetes self-management education. Support for Ethiopian patients with diabetes is also recommended.

Support needed related positively with QOL and diabetes impact. Even though the support received did not relate to QOL, it related positively with satisfaction and diabetes-related worry. These findings are supported by some previous studies [42–44]. Ramkisson, Pillay [45] reported that social support enhances life satisfaction and minimizes diabetes-related worry. Psychological distress is common in people with diabetes [46]; as a result, they need social support [43] to enhance their confidence in performing self-care [44]. Good diabetes management and self-care improve glycemic control [3], and fear of hypoglycemia associated with QOL [47]. Hence, continuous social support may have attributed to improved QOL.

Meanwhile, support attitude was not associated with overall QOL but was negatively associated with satisfaction with diabetes life. People with diabetes have a good support attitude to facilitate their interactions with other people in managing their disease [1]. The reason for the current contradictory finding can be personal expectations, particularly about medical management and outcomes, and available social support [3], and QOL depends on personal perceived ability and efficacy of self-management practice to control diabetes-related outcomes [48].

Attending tertiary education was related positively with diabetes-related worry, and this finding is inconsistent with the findings of previous studies [12,49]. Good education can enhance the functional capacities of people with diabetes [50]. The association between education and diabetes-related worry found in the current study might be due to the fact that education is associated with high diabetes knowledge [51]; hence, low worry due potential complications related to diabetes was observed in the present study. Comorbidity with hypertension was related with low satisfaction. Rodríguez-Almagro, García-Manzanares [12] reported that hypertension worsened social or vocational worry and decreased satisfaction in people with diabetes. A possible explanation for the decreased satisfaction is that hypertension is one of the most common and frequently coexisting factors related to type 2 diabetes [3,49]. Hence the participants might have low satisfaction because of hypertension. Given that comorbidity with hypertension was not related with impact, social or vocational worry, and diabetes-related worry in people with type 2 diabetes, hypertension prevention and early management are recommended to improve patient’s satisfaction. Patients who were separated or divorced related negatively quality of life compared with those married. This finding is consistent with the study from Ethiopia [20], which reported that patients who were married had high QOL. The possible explanation for the negatively related with quality of life was that those who separated or divorced may had low life satisfaction, felt high impact, or had high worry about the disease. Compared with females, male patients with diabetes related positively with QOL. These results are consistent with those of previous studies [20,40,52,53]. Females are more likely to have problems completing usual activities [45] and worry more about their diabetes due to increased risk of complications [54,55]. These findings suggest that females with diabetes need more support on social or vocational issues. Another possible reason is that females are more concerned about the disease and may be involved less in diabetes management and self-care activities. Age was negatively related with quality of life and all its domains. The current study found that as age increases, the quality of life decreases. This finding is congruent with the study conducted in Ethiopia [20] and may be attributed to the fact that diabetes increases with age [56]. This attitude may decrease as they age. Another possible reason is that as age increase, diabetes-related complications and pain increases, and physical activities may be limited. Hence, support for older patients with diabetes is necessary to improve their quality of life.

### Limitation of the study

A convenience sampling technique, recruitment of subjects from a single hospital and recruitment of subjects those speaking only Afaan Oromoo were limitations of the study.

## Conclusions

Being male, homemakers, self-management practice, and support needed were positively predicted quality of life, whereas older age, separation or divorce from a partner, and long diabetes life negatively predicted quality of life in adults with type 2 diabetes in western Ethiopia. Thus, encouraging self-management practice by diabetic patients and continuous family or friend support from their families or friends are necessary to enhancing quality of life among people with type 2 diabetes.

## Supporting information

S1 Dataset (SAV)
